# Alterations in tissue oxygen saturation measured by near-infrared spectroscopy in trauma patients after initial resuscitation are associated with occult shock

**DOI:** 10.1007/s00068-022-02068-w

**Published:** 2022-09-02

**Authors:** Andrea Campos-Serra, Jaume Mesquida, Sandra Montmany-Vioque, Pere Rebasa-Cladera, Marta Barquero-Lopez, Ariadna Cidoncha-Secilla, Núria Llorach-Perucho, Marc Morales-Codina, Juan Carlos Puyana, Salvador Navarro-Soto

**Affiliations:** 1 Department of Surgery, Universitat Autònoma de Barcelona, Parc Taulí Hospital Universitari, Parc del Taulí 1, 08208 Sabadell (Barcelona), Spain; 2 Critical Care Department, Parc Taulí Hospital Universitari, Sabadell, Spain; 3 Anesthesiology Department, Parc Taulí Hospital Universitari, Sabadell, Spain; 4 Critical Care Department, Hospital Universitari Josep Trueta, Girona, Spain; 5 Department of Surgery, University of Pittsburgh, Pittsburgh, USA

**Keywords:** Trauma, Occult shock, Microcirculation, Hemodynamics, Resuscitation

## Abstract

**Purpose:**

Persistent occult hypoperfusion after initial resuscitation is strongly associated with increased morbidity and mortality after severe trauma. The objective of this study was to analyze regional tissue oxygenation, along with other global markers, as potential detectors of occult shock in otherwise hemodynamically stable trauma patients.

**Methods:**

Trauma patients undergoing active resuscitation were evaluated 8 h after hospital admission with the measurement of several global and local hemodynamic/metabolic parameters. Apparently hemodynamically stable (AHD) patients, defined as having SBP ≥ 90 mmHg, HR < 100 bpm and no vasopressor support, were followed for 48 h, and finally classified according to the need for further treatment for persistent bleeding (defined as requiring additional red blood cell transfusion), initiation of vasopressors and/or bleeding control with surgery and/or angioembolization. Patients were labeled as “Occult shock” (OS) if they required any intervention or “Truly hemodynamically stable” (THD) if they did not. Regional tissue oxygenation (rSO_2_) was measured non-invasively by near-infrared spectroscopy (NIRS) on the forearm. A vascular occlusion test was performed, allowing a 3-min deoxygenation period and a reoxygenation period following occlusion release. Minimal rSO_2_ (rSO_2_min), Delta-down (rSO_2_–rSO_2_min), maximal rSO_2_ following cuff-release (rSO_2_max), and Delta-up (rSO_2_max–rSO_2_min) were computed. The NIRS response to the occlusion test was also measured in a control group of healthy volunteers.

**Results:**

Sixty-six consecutive trauma patients were included. After 8 h, 17 patients were classified as AHD, of whom five were finally considered to have OS and 12 THD. No hemodynamic, metabolic or coagulopathic differences were observed between the two groups, while NIRS-derived parameters showed statistically significant differences in Delta-down, rSO_2_min, and Delta-up.

**Conclusions:**

After 8 h of care, NIRS evaluation with an occlusion test is helpful for identifying occult shock in apparently hemodynamically stable patients.

**Level of evidence:**

IV, descriptive observational study.

**Trial registration:**

ClinicalTrials.gov Registration Number: NCT02772653.

## Introduction

Trauma is the most frequent cause of death among people aged between 5 and 29 years old worldwide [1], and in trauma patients, hemorrhagic shock is the first cause of preventable death [2, 3]. To increase the chances of survival, efforts must be made to rapidly detect and resuscitate patients from shock [2, 4, 5]. The classical definition of shock is the alteration of vital signs, but, after normalization of these parameters, up to 85% of severely injured trauma patients still have persistent hypoperfusion and ongoing tissue acidosis, also known as occult shock, which may lead to organ dysfunction and death [4, 6]. Traditional markers such as blood pressure, heart rate, urine output, and mental status are still commonly used to guide resuscitation in trauma patients [7, 8] but they are non-specific [9, 10]. Other markers such as arterial lactate or base deficit (BD) are accurate and objective but require invasive monitoring or intermittent blood sampling, and may still fail to detect regional hypoperfusion [9]. To improve the ability to detect ongoing hypoperfusion, monitoring of regional “non-vital” areas, such as the splanchnic area or peripheral skeletal muscle, has been proposed [11, 12]. It has been suggested that regional hypoperfusion should be evaluated at the end of conventional resuscitation, when “global” hypoperfusion markers have been corrected [11]. On the whole, even though several markers of resuscitation have been described, to date, no gold standard has been identified [4].

During hemorrhagic shock, the activation of the sympathetic nervous system causes a redistribution of the blood flow from the periphery to the central compartment, to maintain optimal perfusion of the vital organs [13]. Near-infrared spectroscopy (NIRS) provides a rapid, noninvasive, and continuous estimate of local tissue oximetry, also known as regional tissue oxygen saturation (rSO_2_), and it is generally used in peripheral areas such as the skeletal muscle [9, 14, 15]. Real-time measurements of rSO_2_ allow dynamic assessment of the patient’s response to resuscitation, and additional data can be obtained from a vascular occlusion test (VOT), a regional stress test that has repeatedly demonstrated its value in the assessment of hemodynamic alterations in several scenarios [10, 14].

The objective of this study was to establish whether NIRS-derived muscle rSO_2_, and the response to a VOT, were associated with occult shock once conventional global resuscitation was considered to be complete. To this end, other commonly used markers of shock were compared, including vital signs, Shock Index, ROPE index, hemoglobin, natriuretic atrial peptide, arterial BD, serum lactate concentration, and coagulopathy, defined according to rotational thromboelastography (ROTEM^®^).

## Methods

### Design and setting

We conducted a prospective observational study at a university hospital (Parc Taulí University Hospital, Sabadell, Spain). The local Ethics Committee (Comitè Ètic d'Investigació Clínica, Institut d’Investigació i Innovació Parc Taulí I^3^PT (Reference 2,016,529) approved the study and it was registered at Clinicaltrials.gov (Reference NCT02772653). Informed consent was obtained from each patient or each patient’s next of kin. This study is presented following the STROBE recommendations for reporting observational studies [16].

### Patients

Severely injured trauma patients with physiological or anatomical prehospital triage criteria (Table 1) according to ATLS [8] were included.

**Table 1:** Physiological and anatomical prehospital triage criteria

Physiological criteria	Anatomical criteria
Systolic blood pressure < 90 mmHg	All penetrating injuries to head, neck, torso and extremities proximal to the elbow and knee
Respiratory rate < 10 or > 29 breaths/min	Chest wall instability or deformity
Glasgow coma scale score ≤ 13	Two or more proximal long-bone fractures
Non-palpable peripheric pulses	Crushed, degloved mangled or pulseless extremity
	Amputation proximal to wrist or ankle
	Pelvic fractures

Exclusion criteria were: age under 16 years old, patients transferred from/to other hospitals within 24 h of the accident, patients with isolated neurological injury, and the impossibility of measuring NIRS-derived tissue oxygenation parameters due to local conditions such as trauma in both upper limbs, and skin and/or vascular injuries affecting the thenar eminence.

### Protocol

Resuscitation markers were evaluated in patients undergoing active resuscitation after 8 h of hospital care. In critical care, although it is accepted that “the earlier, the better”, this initial window for achieving resuscitation goals is usually set at 6–8 h [17]. In our study, we aimed at analyzing the ability of regional tissue oxygenation to detect occult hypoperfusion once the initial resuscitation process would be ideally complete. Therefore, we chose the 8-h time frame as a sufficient window to complete this initial resuscitation. Patients with SBP ≥ 90 mmHg, HR < 100 bpm and no vasopressor support were defined as apparently hemodynamically stable (AHD), as opposed to hemodynamically unstable (HDU) when at least one of the criteria was not met. AHD patients were later categorized into two groups according to the need for additional treatment between the eighth and the 48th hour after the injury: AHD patients were finally labeled as having “Occult shock” (OS) if they needed further treatment for persistent bleeding, defined as requiring additional transfusion, initiation of vasopressors, or needing surgery or angioembolization. Patients who did not need any additional treatment were finally labeled as “truly HD stable” (THD). It should be taken into consideration that elective surgical interventions were not considered to be interventions for bleeding control. On the other hand, patients who had semi-elective surgical interventions between the 8th and the 48th hours after the accident had no active bleeding that needed surgical control and did not need any red blood cell transfusion (RBCT) nor vasopressors administration, which could have been considered a cause of bias interpreting the results of this study.

Hemodynamic, metabolic, coagulopathy, and microcirculatory parameters were measured simultaneously.

### Measured variables

1. Hemodynamic variables included: systolic blood pressure (SBP), diastolic blood pressure (DBP), heart rate (HR), Shock Index (SI) [HR/SBP] and ROPE Index [HR/(SBP-DBP)].
2. Blood samples included: hemoglobin, natriuretic atrial peptide (NAP), base deficit and serum lactate concentration.
3. Coagulopathy was assessed with ROTEM^®^ and classified in five phenotypes, according to the degree of abnormality observed: 0 = normal; 1 = fibrinogen deficiency; 2 = hypocoagulability; 3 = platelet deficiency; 4 = global deficiency; 5 = global deficiency and hyperfibrinolysis.
4. Regional oxygen saturation (rSO_2_) was recorded continuously using the INVOS™ 5100C Cerebral/Somatic Oximeter (Medtronic, Essex, UK). The rSO_2_ 15 mm optical surface probe was placed on intact skin on the forearm muscle. In addition to the steady-state rSO_2_ value, the response to a transient ischemic challenge was also computed. The ischemic challenge consisted in a standardized Vascular Occlusion Test (VOT), and was performed as previously described in the literature [18]. Briefly, a blood pressure cuff was placed on the arm and rapidly inflated at 50 mmHg above systolic pressure and kept inflated for a three-minute period. Then, the cuff was rapidly deflated and the minimum rSO_2_ (rSO_2_min) and maximum rSO_2_ (rSO_2_max) values were recorded. VOT-derived variables included Delta-down (difference between basal rSO_2_ and rSO_2_min) and Delta-up (difference between rSO_2_min and rSO_2_max). Absolute rSO_2_ and VOT-derived variables were obtained using the INVOS Analytics Tool v1.2 (Medtronic, Essex, UK).

Patient demographics, prehospital triage criteria, Injury Severity Score (ISS), mechanism of injury, causes of death, vasopressor administration, blood products, and operative and interventional radiology procedures were recorded.

### Normal data set with healthy volunteers

To determine the normal range of forearm muscle rSO_2_ and the normal response to VOT, forearm muscle rSO_2_ and VOT readings were taken from healthy volunteers whose age and sex were similar to the patients analyzed for this study. Generally, these individuals were hospital and university staff and medical students. Additional parameters recorded included individual’s age, sex, blood pressure, and comorbidities. Exclusion criteria were consumption of caffeine within the previous 8 h before the test, taking medications with cardiovascular effects, and/or previous known peripheral vascular disease.

### Statistical analysis

Statistical analysis was performed by means of IBM SPSS statistics v25 software (SPSS Inc, Chicago, IL, USA). The normal distribution of the variables studied was confirmed using the Kolmogorov–Smirnov test. Accordingly, continuous variables were expressed as means ± standard deviation (SD), and categorical variables were expressed as absolute numbers and proportions (%). A descriptive analysis was performed. Differences between groups were assessed using the Chi-squared test for categorical variables, and the Kruskal–Wallis test, Mann–Whitney’s *U*-test or Student’s *t*-test for continuous variables, as appropriate. A two-tailed *p* value of less than 0.05 was taken to indicate statistical significance.

## Results

### Healthy volunteers

From December 2019 to March 2020, basal rSO_2_ and VOT-derived variables were obtained from 48 healthy volunteers. Main characteristics of the sample population and NIRS-related results are shown in Table 2.

**Table 2:** Demographics, and NIRS-related results of healthy volunteers

*N*	48
Age (years)	43 ± 16
Sex Male (*n*, %) Female (*n*, %)	37 (77%) 11 (23%)
Absolute rSO_2_ (%)	71 ± 7
Minimum rSO_2_ (%)	43 ± 15
Maximum rSO_2_ (%)	86 ± 8
Delta-down (%)	28 ± 12
Delta-up (%)	43 ± 16

*NIRS* near-infrared spectroscopy, *rSO*_*2*_ regional oxygen saturation

### Trauma patients

**Fig. 1:**
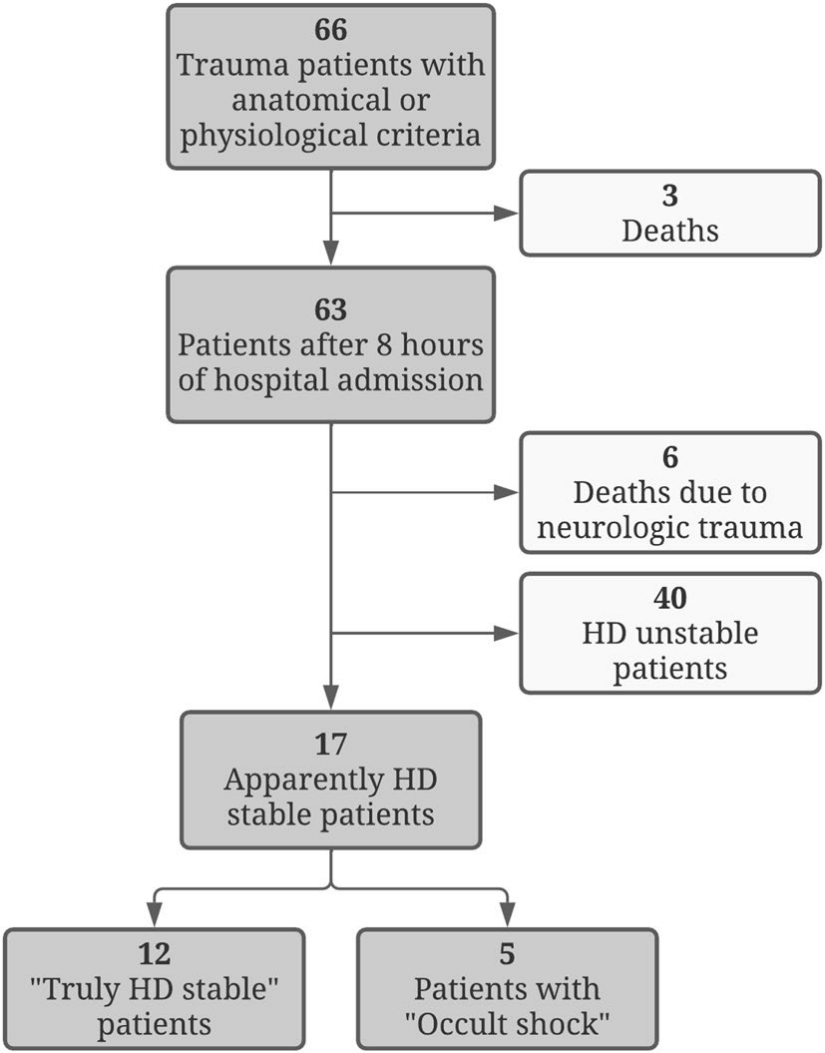
Flow chart illustrating the study recruitment and the final categorization of apparently HD stable patients

From May 2016 to March 2019, 66 consecutive trauma patients with eligible criteria for this study were admitted to our hospital. Mean age was 42 ± 15 years old, 80% were males and median injury severity score (ISS) was 25.5 (IQR 8–68). The characteristics of AHD and HDU patients are shown in Table 3. Three patients (5%) died within the initial hours of admission, and six patients (9%) died of severe brain injury later in the course of the initial care and were excluded from the final analysis. After 8 h of hospital admission, 40 patients were considered to be HDU, (37 patients had a HR ≥ 100 bpm; 9 patients had a SBP < 90 mmHg and 32 patients were under vasopressor support) and 17 patients were classified as AHD (Fig. 1). In the HDU group, three patients died after 8 h of hospital admission (7.5%) while in the AHD group there were no deaths. After 48 h of follow-up, five of the 17 AHD patients were classified as OS: three patients needed RBCT, one needed vasoactive drug administration plus RBCT, and one patient needed surgery to control persistent bleeding plus RBCT. The patient with persistent bleeding needing surgery had a penetrating injury to the stomach with persistent bleeding of the posterior gastric wall, he presented hematemesis in the 14th hour of hospital stay and was re-operated in the 16th hour. None of the OS patients did undergo any semi-elective surgical intervention between the 8th and 48th hour. On the contrary, two patients of the THS group did undergo semi-elective surgical interventions between the 8th and 48th hour, but neither needed surgical bleeding control, RBCT nor vasopressor administration: one had a laparoscopic exploration of a penetrating diaphragmatic injury needing suture and the other was operated for an open abdomen closure.

**Table 3:** Main characteristics of AHD and HDU trauma patients

	AHD	HDU	*p*
*N*	17	40	
Age (years) (mean ± SD)	41 ± 17	43 ± 13	0.6
Sex male (*n*, %)	14 (82%)	35 (88%)	0.5
Prehospital physiological (*n*, %) criteria anatomical (*n*, %)	6 (35%) 11 (65%)	30 (75%) 10 (25%)	0.004
Mechanism blunt (*n*, %) penetrating (*n*, %)	14 (82%) 3 (18%)	36 (90%) 4 (10%)	0.4
ISS (mean ± SD)	17 ± 8	29 ± 13	< 0.001

Comparison between healthy volunteers and trauma patients is presented in Table 4. Age and gender of healthy volunteers and trauma patients did not show significant differences. With regard to NIRS-related variables included in the protocol, there were no statistically significant differences between healthy volunteers and trauma patients, except for lower rSO_2_-max values in trauma patients (86 ± 8 vs 80 ± 7, *p* 0.02). No differences between volunteers and THD were observed, while OS patients showed alterations in rSO_2_-min, Delta-down, and Delta-up as compared to healthy controls. HDU only differed from healthy subjects in terms of their rSO_2_-max following the VOT.

**Table 4:** Comparison of variables between healthy volunteers and trauma patients

	Healthy volunteers	Trauma patients	HDU	AHD	
				THD	Occult shock
	*N* = 48	*N* = 57	*N* = 40	*N* = 12	*N* = 5
	Mean ± SD	Mean ± SD	Mean ± SD	Mean ± SD	Mean ± SD
NIRS absolute rSO_2_	71 ± 7	68 ± 8	68 ± 8	68 ± 10	72 ± 11
NIRS delta-down	28 ± 12	31 ± 13	31 ± 12	23 ± 12	48 ± 11*
NIRS delta-up	43 ± 16	43 ± 13	43 ± 16	35 ± 17	60 ± 10
NIRS rSO_2_ min	43 ± 15	37 ± 16	36 ± 18	45 ± 13	24 ± 2**
NIRS rSO_2_ max	86 ± 8	80 ± 7**	79 ± 4**	80 ± 11	84 ± 10

Mann–Whitney *U*-test for comparisons between healthy volunteers and trauma patients

*NIRS* near-infrared spectroscopy, *HDU* hemodynamically unstable, *AHD* apparently hemodynamic stable, *THD* truly hemodynamically stable

^*^p < 0.05, **p < 0.01

The Kruskal–Wallis test showed different distributions of the NIRS-related variables among the three trauma groups. While no significant differences were detected between HDU and THD patients, OS patients differed from the rest of the groups (Table 4).

Table 5 shows the relationship between all the resuscitation markers analyzed and the identification of occult shock in AHD stable trauma patients. Only NIRS VOT-derived variables showed statistically significant differences between THDs and OS patients.

**Table 5:** Associations between resuscitation markers and occult shock after 8 h of hospital admission

	Truly HD stable	Occult shock	
	*N* = 12 (70%)	*N* = 5 (30%)	
	Mean ± SD	Mean ± SD	*p*
Heart rate (bpm)	80 ± 10.6	82 ± 12	0.7
Systolic blood pressure (mmHg)	118 ± 18.7	115 ± 13.6	0.7
Shock index	0.69 ± 0.2	0.72 ± 0.1	0.7
ROPE index	1.6 ± 0.6	2.2 ± 1.3	0.2
Hemoglobin (g/L)	110 ± 17.4	94 ± 0.85	0.2
Serum lactate (mg/dL)	21.4 ± 12.1	25.8 ± 9.0	0.5
Base deficit (mmol/L)	-1.5 ± 2.0	-0.5 ± 1.5	0.4
Natriuretic atrial peptide (nmol/L)	2.4 ± 0.8	6.7 ± 8.0	0.3
ROTEM coagulopathy	0.5 ± 0.8	0.2 ± 0.5	0.5
NIRS absolute rSO_2_	68.4 ± 9.6	72.0 ± 11.1	0.6
NIRS delta-down	23.3 ± 11.5	48.3 ± 10.5	**0.016**
NIRS delta-up	34.7 ± 16.8	60.3 ± 9.5	**0.047**
NIRS rSO_2_ min	44.8 ± 13.1	23.7 ± 1.5	**0.010**
NIRS rSO_2_ max	79.5 ± 11.3	84.0 ± 10.1	0.1

Bold values indicate statistical significance of *p* values of less than 0.05

*HD* hemodynamically, *NIRS* near-infrared spectroscopy, *rSO*_*2*_ Regional oxygen saturation

## Discussion

Our data suggest that, after 8 h of active treatment, alterations in rSO_2_-related parameters may help to detect apparently hemodynamically stable patients who are still under-resuscitated. These findings are relevant, since alterations in NIRS parameters can provide clinicians with important information regarding the evaluation of otherwise clinically stable patients.

Occult shock, defined as persistent hypoperfusion with normal vital signs, remains a controversial entity [5], and its definition has evolved along with the appearance of new metabolic and perfusion parameters. In recent years, regional parameters have demonstrated their additional value compared with global metabolic parameters [5, 12, 19, 20] such as lactate, thus further stressing the importance of the concept of occult shock. In the present study, occult shock was defined as the need for additional blood cell transfusion, use of vasopressors or need for surgery or angioembolization in trauma patients, whose vital signs were normal, between the 8th and 48th hour of hospital admission. Our results suggest that regional tissue oxygenation parameters play a key role in the detection of occult shock and in the need for further resuscitation interventions.

Our findings are consistent with previous data on NIRS monitoring, and are based on a strong pathophysiological rationale. We observed that, in apparently stable patients, higher rates of desaturation following a VOT were associated with the need for further resuscitation interventions to control bleeding in the following 48 h. These higher rSO_2_ desaturation rates can only be attributed to two underlying mechanisms, or a combination of them: (a) diminished local blood flow, and (b) increased local metabolic rate. In situations of hypovolemia, blood flow is diverted from the periphery to the central compartment, causing a “stealing effect” of blood from these peripheral or non-vital areas. This compensatory mechanism is mainly driven by the activation of the sympathetic system, which also increases the metabolic rate, as a result of the effect of the release of catecholamines [13]. Regrettably, our study does not allow us to separate these two phenomena, but our results are consistent with those of other authors who have shown that alterations in VOT-derived parameters are associated with the redistribution of the blood flow towards the central compartment and sympathetic activation [19, 20]. Interestingly, VOT-derived parameters are useful in situations where the compensatory mechanisms are subclinical and are not detected by the standard hemodynamic monitoring tools.

Previous studies have already described NIRS technology as a useful tool for detecting tissue hypoperfusion in trauma patients [4, 14, 15, 21, 22]. The results of our study coincide with those of Guyette et al. [10] which obtained a prehospital absolute rSO_2_ and performed a VOT in 150 trauma patients, and concluded that, even though rSO_2_ had prognostic value for mortality and multiple organ failure, it did not identify occult shock states. This was considered an inherent limitation of the technology, insofar as peripheral vasoconstriction associated with compensated shock states may be accompanied by a reduction in regional tissue oxygen demand and compromised supply. However, stressing the system by producing a vascular occlusion yielded parameters such as the rSO_2_ de-saturation and re-saturation slopes, which distinguished between patients requiring bleeding control maneuvers and those that did not. Additionally, the analysis of lactate and SBP found no statistical relationship for these variables, and the authors concluded that VOT-derived variables were capable of detecting occult shock when vital signs and lactate were normal. NIRS technology has also been recommended in AHD patients before starting resuscitation maneuvers [15] or when “global” resuscitation maneuvers have finished, to identify patients with persistent microcirculatory hypoperfusion [14, 21]. The second case coincides with the moment when occult shock was evaluated in this study, after 8 h of hospital admission. Conversely, Crookes et al. [9] found a statistically significant relationship only between absolute rSO_2_ and severe shock, and not with mild or moderate shock. It should be stressed that no VOT was performed; therefore, in situations of severe shock absolute rSO_2_ is altered, but in states of moderate or occult shock it remains normal while VOT-derived variables are altered. In general, we can infer that absolute rSO_2_ values may be capable of detecting overt shock, and that VOT-derived parameters are useful in situations where the hemodynamic status is apparently compensated and provide additional help in the detection of tissue hypoperfusion. When analyzing the HDU group, one would expect HDU patients to show lower regional oxygenation parameters. However, the lack of differences might derive from our definition of HDU, including those patients who were hypotensive, tachycardic, and/or requiring vasopressors. In fact, 27 patients were classified as HDU because they were on vasopressors. Therefore, although we considered those patients to be HDU, they were probably resuscitated, with normalized tissue perfusion/oxygenation, despite requiring vasopressors to achieve that normalization. Our study adds to the current evidence and underlines the value of regional tissue oxygenation as part of the monitoring tool kit for the hemodynamic resuscitation of trauma patients.

The predictive power of non-NIRS-related variables was very limited except for lactate and BD, two parameters that have classically been related to occult shock detection [4–6, 23–26]. However, some publications such as James et al. [27] reject the relationship between tissue acidosis and lactate, considering that lactate elevation is related to the catecholaminergic response to trauma. Other authors such as Pal et al. [23] and Petrosoniak et al. [5] have associated persistent elevation of lactate with occult shock; Abramson et al. [24] considered that patients with pathologic lactate and normalized vital signs needed more aggressive monitoring. Brohi et al. [25] stated that lactate and BD can be considered equivalent and that lactate alteration is an indicator of hypoperfusion, while Guyette et al. [26] recommend prehospital lactate evaluation to identify occult shock in patients with normalized vital signs.

Blow et al. [6] defined occult shock as the presence of lactate > 25 mg/dL in patients with SBP > 100 mmHg, HR < 120 mmHg and diuresis > 1 ml/kg/h. Patients who met the inclusion criteria received intensive resuscitation to normalize lactate values during the first 24 h of hospital stay, and showed reduced mortality when normalization was achieved.

In contrast, our study did not detect a statistically significant relationship between lactate or BD and occult shock. This absence of a statistical relation might be attributable, on the one hand, to the small sample size, as we compared 12 THD patients with five OS patients. On the other hand, most authors associate lactate and BD with mortality [4, 6, 24, 25, 28] and multiple organ failure [4, 6, 25, 28] considering it to be secondary to tissue hypoperfusion; however, in our study, NIRS VOT-derived variables showed a higher capacity to detect microcirculation alterations, even though the sample of patients analyzed was small.

A previous study by our group considered the Shock Index (SI) as a marker of occult shock when it was ≥ 0.8 [29] because patients subsequently needed maneuvers to control surgical bleeding or activation of a massive transfusion protocol. It has also been considered as an occult shock marker with scores ≥ 0.9 [30, 31], in relation to multiple organ failure or mortality in patients with previously normal vital signs.

In contrast, our study did not find a statistically significant relationship between the SI and occult shock. This may have been due to the moment in time when the SI was evaluated. Generally, the studies that associate the SI with bleeding control maneuvers use it during prehospital care [31, 32] or at hospital admission [29, 31, 33, 34]; no studies have evaluated the SI after the first hour of hospital admission.

The main limitation of our study is the small sample of trauma patients analyzed. However, and though the number of patients is small, VOT-derived variables presented statistically significant relationships with occult shock, while the other variables did not.

## Conclusions

After 8 h of hospital care, NIRS evaluation with an occlusion test might be helpful for identifying occult shock in otherwise apparently hemodynamically stable trauma patients. In this study, no other resuscitation marker analyzed showed a statistically significant association with occult shock detection. Therefore, VOT-derived parameters may be a relevant screening tool for detecting patients at risk of hemodynamic deterioration. Further and larger prospective study would be needed to confirm our findings.

## Data Availability

Available upon request.

## Code availability

Available upon request.

## References

[1] World Health organization. Global Health Observatory. Top 10 causes of death in 2016. https://www.who.int/gho/mortality_burden_disease/causes_death/top_10/en/.

[2] O’Reilly D, Mahendran K, West A, Shirley P, Walsh M, Tai N. Opportunities for improvement in the management of patients who die from haemorrhage after trauma. Br J Surg. 2013;100(6):749–55. 10.1002/bjs.9096. 10.1002/bjs.9096 23483534

[3] Pfeifer R, Tarkin IS, Rocos B, Pape HC. Patterns of mortality and causes of death in polytrauma patients-Has anything changed? Injury. 2009;40(9):907–11. 10.1016/j.injury.2009.05.006. 10.1016/j.injury.2009.05.006 19540488

[4] Tisherman SA, Barie P, Bokhari F, Bonadies J, Daley B, Diebel L, et al. Clinical practice guideline: endpoints of resuscitation. J Trauma. 2004;57(4):898–912. 10.1097/01.TA.0000133577.25793.E5. 10.1097/01.ta.0000133577.25793.e5 15514553

[5] Petrosoniak A, Hicks C. Resuscitation resequenced: a rational approach to patients with trauma in shock. Emerg Med Clin N Am. 2018;36(1):41–60. 10.1016/j.emc.2017.08.005. 10.1016/j.emc.2017.08.005 29132581

[6] Blow O, Magliore L, Claridge JA, Butler K, Young J. The golden hour and the silver day: detection and correction of occult hypoperfusion within 24 hours improves outcome from major trauma. J Trauma. 1999;47(5):964–9. 10.1097/00005373-199911000-00028. 10.1097/00005373-199911000-00028 10568731

[7] Bruijns SR, Guly HR, Bouamra O, Lecky F, Lee WA. The value of traditional vital signs, shock index, and age-based markers in predicting trauma mortality. J Trauma Acute Care Surg. 2013;74(6):1432–7. 10.1097/TA.0b013e31829246c7. 10.1097/TA.0b013e31829246c7 23694869

[8] ATLS (2018). Advanced trauma life support. 10th edition. Chicago: committee on trauma.

[9] Crookes BA, Cohn SM, Bloch S, Amortegui J, Manning R, Li P, et al. Can near-infrared spectroscopy identify the severity of shock in trauma patients? J Trauma. 2005;58(4):806–16. 10.1097/01.TA.0000158269.68409.1C 10.1097/01.ta.0000158269.68409.1c 15824660

[10] Guyette F, Gomez H, Suffoletto B, Quintero J, Mesquida J, Kim H, et al. Prehospital dynamic tissue oxygen saturation response predicts in-hospital lifesaving interventions in trauma patients. J Trauma Acute Care Surg. 2012;2(4):930–5. 10.1097/TA.0b013e31823d0677. 10.1097/TA.0b013e31823d0677 PMC3770128 22491607

[11] Mesquida J, Masip J, Gili G, Artigas A, Baigorri F. Thenar oxygen saturation measured by near infrared spectroscopy as a noninvasive predictor of low central venous oxygen saturation in septic patients. Intensive Care Med. 2009;35(6):1106–9. 10.1007/s00134-009-1410-y. 10.1007/s00134-009-1410-y 19183952

[12] De Backer D, Ospina-Tascon G, Sagado D, Favory R, Creteur J, Vincent JL. Monitoring the microcirculation in the critically ill patient: current methods and future approaches. Intensive Care Med. 2010;36(11):1813–25. 10.1007/s00134-010-2005-3. 10.1007/s00134-010-2005-3 20689916

[13] Bonanno FG. Physiopathology of shock. J Emerg Trauma Shock. 2011;4(2):222–32. 10.4103/0974-2700.82210. 10.4103/0974-2700.82210 PMC3132363 21769210

[14] Mesquida J, Gruartmoner G, Espinal C. Skeletal muscle oxygen saturation (StO2) measured by near-infrared spectroscopy in the critically ill patients. Biomed Res Int. 2013;2013:1–8. 10.1155/2013/502194. 10.1155/2013/502194 PMC3763593 24027757

[15] Cohn SM, Nathens AB, Moore FA, Rhee P, Puyana JC, Moore EE, et al. Tissue oxygen saturation predicts the development of organ dysfunction during traumatic shock resuscitation. J Trauma. 2007;62(1):44–54. 10.1097/TA.0b013e31802eb817. 10.1097/TA.0b013e31802eb817 17215732

[16] Von Elm E, Altman DG, Egger M, Pocock SJ, Gøtzsche PC, Vandenbroucke JP. Declaración de la iniciativa STROBE (strengthening the reporting of observational studies in epidemiology): directrices para la comunicación de estudios observacionales. Rev Esp Salud Publica. 2008;82(3):251–9. 10.1157/13119325. 10.1590/s1135-57272008000300002 18711640

[17] Ferrer R, Artigas A, Levy MM, Blanco J, González-Díaz G, Garnacho-Montero J, et al. Improvement in process of care and outcome after a multicenter severe sepsis educational program in Spain. JAMA. 2008;299(19):2294–303. 10.1001/jama.299.19.2294 10.1001/jama.299.19.2294 18492971

[18] Mayeur C, Campard S, Richard C, Teboul JL. Comparison of four different vascular occlusion tests for assessing reactive hyperemia using near-infrared spectroscopy. Crit Care Med. 2011;39(4):695–701. 10.1097/CCM.0b013e318206d256 10.1097/CCM.0b013e318206d256 21220999

[19] Mesquida J, Gruartmoner G, Espinal C, Masip J, Sabatier C, Villagrá A, et al. Thenar oxygen saturation (StO2) alterations during a spontaneous breathing trial predict extubation failure. Ann Intensiv Care. 2020;10(1):54. 10.1186/s13613-020-00670-y. 10.1186/s13613-020-00670-y PMC7214564 32394211

[20] Gruartmoner G, Mesquida J, Masip J, Martínez ML, Villagra A, Baigorri F, et al. Thenar oxygen saturation during weaning from mechanical ventilation: an observational study. Eur Respir J. 2014;43(1):213–20. 10.1183/09031936.00126312. 10.1183/09031936.00126312 23314894

[21] Gruartmoner G, Mesquida J, Baigorri F. Saturación tisular de oxígeno en el paciente crítico. Med Intensiva. 2014;38(4):240–8. 10.1016/j.medin.2013.07.004. 10.1016/j.medin.2013.07.004 24035697

[22] McKinley BA, Marvin RG, Cocanour CS, Moore FA. Tissue hemoglobin O2 saturation during resuscitation of traumatic shock monitored using near infrared spectrometry. J Trauma. 2000;48(4):637–42. 10.1097/00005373-200004000-00009. 10.1097/00005373-200004000-00009 10780595

[23] Pal JD, Victorino GP, Twomey P, Liu TH, Bullard MK, Harken AH, et al. Admission serum lactate levels do not predict mortality in the acutely injured patient. J Trauma. 2006;60(3):583–9. 10.1097/01.ta.0000205858.82575.55. 10.1097/01.ta.0000205858.82575.55 16531858

[24] Abramson D, Scalea T, Hitchcock R, Trooskin S, Henry S, Greenspan J. Lactate clearance and survival following injury. J Trauma. 1993;35(4):584–9. 10.1097/00005373-199310000-00014 10.1097/00005373-199310000-00014 8411283

[25] Brohi K, Cohen MJ, Ganter MT, Matthay MA, Mackersie RC, Pittet JF. Acute traumatic coagulopathy: initiated by hypoperfusion: modulated through the protein C pathway? Ann Surg. 2007;245(5):812–8. 10.1097/01.sla.0000256862.79374.31. 10.1097/01.sla.0000256862.79374.31 PMC1877079 17457176

[26] Guyette F, Suffoletto B, Castillo J-L, Quintero J, Callaway C, Puyana J-C. Prehospital serum lactate as a predictor of outcomes in trauma patients: a retrospective observational study. J Trauma. 2011;70(4):782–6. 10.1097/TA.0b013e318210f5c9. 10.1097/TA.0b013e318210f5c9 21610386

[27] James JH, Luchette FA, McCarter FD, Fischer JE. Lactate is an unreliable indicator of tissue hypoxia in injury or sepsis. Lancet. 1999;354(9177):505–8. 10.1016/S0140-6736(98)91132-1. 10.1016/S0140-6736(98)91132-1 10465191

[28] Montmany Vioque S, Navarro Soto S, Rebasa Cladera P, Luna Aufroy A, Gómez Díaz C, Llaquet BH. Medición del ácido láctico en pacientes politraumatizados y su utilidad como factor predictor de mortalidad y fallo multiorgánico. Cir Esp. 2012;90(2):107–13. 10.1016/j.ciresp.2011.07.011. 10.1016/j.ciresp.2011.07.011 22206654

[29] Campos-Serra A, Montmany-Vioque S, Rebasa-Cladera P, Llaquet-Bayo H, Gràcia-Roman R, Colom-Gordillo A, et al. The use of the shock index as a predictor of active bleeding in trauma patients. Cir Esp. 2018;96(8):494–500. 10.1016/j.ciresp.2018.04.004. 10.1016/j.ciresp.2018.04.004 29778416

[30] Cannon CM, Braxton CC, Kling-Smith M, Mahnken JD, Carlton E, Moncure M. Utility of the shock index in predicting mortality in traumatically injured patients. J Trauma. 2009;67(6):1426–30. 10.1097/TA.0b013e3181bbf728. 10.1097/TA.0b013e3181bbf728 20009697

[31] Olaussen A, Blackburn T, Mitra B, Fitzgerald M. Review article: shock index for prediction of critical bleeding post-trauma: a systematic review. EMA Emerg Med Australas. 2014;26(3):223–8. 10.1111/1742-6723.12232. 10.1111/1742-6723.12232 24712642

[32] Vandromme MJ, Griffin RL, Kerby JD, McGwin G, Rue LW, Weinberg JA. Identifying risk for massive transfusion in the relatively normotensive patient: utility of the prehospital shock index. J Trauma. 2011;70(2):384–90. 10.1097/TA.0b013e3182095a0a. 10.1097/TA.0b013e3182095a0a 21307738

[33] Loggers SA, Koedam TW, Giannakopoulos GF, Vandewalle E, Erwteman M, Zuidema WP. Definition of hemodynamic stability in blunt trauma patients: a systematic review and assessment amongst Dutch trauma team members. Eur J Trauma Emerg Surg. 2017;43(6):823–33. 10.1007/s00068-016-0744-8. 10.1007/s00068-016-0744-8 PMC5707227 27900417

[34] DeMuro JP, Simmons S, Jax J, Gianelli SM. Application of the shock index to the prediction of need for hemostasis intervention. Am J Emerg Med. 2013;31(8):1260–3. 10.1016/j.ajem.2013.05.027. 10.1016/j.ajem.2013.05.027 23806728

